# Hemorrhage after Tooth Extraction

**Published:** 1883-05

**Authors:** G. Woerner


### HEMORRHAGE AFTER TOOTH EXTRACTION.

G. WOERNER.

It seems that a solution of chloride of iron, to stop dangerous hemorrhage after the extraction of teeth, is regarded with most favor. Indeed, its application is almost always successful, and I may remark, in passing, that an addition of chloride of sodium will make the blood coagulum more solid ; the mixture should be four parts of fluid chloride of iron, two parts of chloride of sodium, and four parts of distilled water.

Such a solution can also be used internally, ten drops in a glass of water, three times daily ; but as I once failed in its application, I would advise my colleagues to use nitrate of silver, which deserves the preference in every respect. Hemorrhages from flabby, diseased gums, from laceration of the gums, etc., can almost immediately be stopped by a good strong cauterization. In cases of hemorrhage from the cavities of the gums, a small piece of nitrate of silver is introduced into the bottom of the cavity, covered with a tampon of cotton, or something similar, and the whole is kept in place by a small clamp, made with very little trouble, out of a good strong watch spring.

If still at hand, the extracted tooth will form a better plug than anything else, if again pressed into the cavity of the gum. The iron cautery with succeeding tamponing, has often been used as the last resource, but in the past, more than at present. No dentist should leave his patient in such case until the hemorrhage is stopped, and then only after very careful instructions, for the danger in not, by any means, over in two or three days. Cases are known where hemorrhage and death by æne-mia occurred after eleven days.

But, aside from the severe hemorrhages, danger may be lurking even in common cases. We often find that weak and delicate persons faint, even during very easy tooth extractions. If we allow the head of the patient to fall back, the blood, saliva, and any possible splinters may accumulate in the posterior part of the buccal cavity. If the patient revives with yawning and labored respiration, the epiglottis raises and the contents of the mouth are drawn into the trachea, which may, in extreme cases, result in suffocation. Care should therefore be taken to give the fainting patient a position which insures a lateral or forward inclination of the head, and to keep the mouth open for a free flow of blood and saliva, and an unobstructed access of air. In conclusion, allow me to call attention to the importance of thoroughly clean instruments, as many evil accidents can be traced to carelessness in this respect.—(*Die Zahntechnische Reform.*)

